# Hydatic Cyst of the Liver

**Published:** 1887-09

**Authors:** 


### HYDATIC CYST OF THE LIVER.

### S. L.

Dr. Tessier publishes in the *Bulletin de la Societe Medicale Hom. de France,* March, 1887, the following interesting case:

A German woman of twenty-seven years entered the Hospital St. Jaques June 11th, feeling for the last two weeks very tired, no appetite, and slow digestion. On the 5th of June she was taken with cramps in the stomach, violent vomiting of bile and food, which kept on during the 6th. On the 7th she had long, lasting chills, colic, vomiting, and black stools. No headache nor nosebleed. We found her with a temperature of 39.5 in the evening, lying on her back and considerably prostrated; pulse small and compressible; tongue with a thick yellow coating; sclerotic yellow. No pain in the right iliac region, no spots on abdomen, severe pain on pressure a little to the right of the xyphoid process, about the edge of the liver. Light bending in of the epigastric hollow. Our anamnesis showed that she cannot digest butter or fats, and that at the age of twelve years she had a decided hollow in the region of the liver.

June 12th–15th.—Lachesis 3d trit., 0.10c.; Sulphate of Quinine 0.75. The prostration and the pains are mitigated by the treatment, but on the 16th colic, and she passes about a dozen hydatic vesicles of the size of a small kernel of a grape. Two or three of these vesicles are intact and inclose other vesicles of the size of a hempseed. June 17th.—Expulsion of a piece of transparent membrane of the size of 0.04 ctm.; epistaxis in the evening. June 18th.—Patient complains of pain on left side, and auscultation reveals light subcrepitant rales at the base. Lachesis continued. June 21st.—Glairy stool, no pains, feels better, though pressure over liver hurts. June 24th.—The stools contain large pieces of transparent membrane and hydatic vesicles of the size of a grape kernel. June 26th.—Sleeps better, wants to eat. Prescription changed to Iodum[1], 5–10 drops, and as she steadily improves this is followed by China[3]. July 10th.—Severe pains in epigastric region, with anguish ; prostration, small filiform pulse and cold extremities, and she passes again for two weeks large pieces of membranes and hydatic vesicles, which are opaque and of a brownish color. July 22d.—An explorative puncture, but only a few drops of blood and pus come out. The pains keep on ; she refuses an operation ; goes back to her own home, where the operation is successfully performed, and after a long and tedious convalescence she regains her health.

Our diagnosis was at first doubtful whether we had to deal with uterus gravis, a hepatitis, or an inflammation of hydatic cyst, but the latter was soon shown up by the discharges. The patient never complained of pain in the right shoulder, never had urticaria, symptoms often witnessed as precursors of hydatids, but she could not bear nor digest butter nor fats.

One of the best articles on hydatids of the liver is found in Harley's *Diseases of the Liver*, page 591, where he says that the parasite usually locates itself in the right lobe of the liver, where it may exist for years without becoming painful, till inflammation sets in. Too often the hydatid fremitus, a particularly tremulous vibratory thrill, cannot be detected, though present, and jaundice may be absent or only appears at the stage when suppuration had set in. Harley recommends a thorough operation for the removal of the entire cyst as preferable to tapping, and many cases of successful issues are on record. Frerichs in his work on *Diseases of the Liver*, III, 21, is of the same opinion, for hydatids, when they have attained a great size, are always a dangerous lesion, and when the hydatids burst into the serous cavities the case almost invariably terminates fatally, and a like result ensues when they burst into the hepatic veins or the vena cava.

In our homœopathic literature we find very little on the treatment of hydatids of the liver, and Tessier deserves great praise for the selection of the simillimum to the totality of the symptoms before a diagnosis could be made out. Lachesis covered the venous affection, the prostration and the suppurative process leading to gangrene of the parts affected, the epistaxes showed the threatening decomposition of the blood, and the cold extremities hinted of collapse. Being the simillimum, no wonder that it acted beneficially, even only as a palliative, but why it was changed to Iodium and China, and thus interfered with in its healing power, is a stigma on the French school, which too often alternates and palliates. Let us have more faith in our homœopathic palliatives and we will have hardly ever a cause where mere lulling pains becomes necessary. It is curious that we find aversion to butter under Arsenicum, Carbo veg., China, and Mercur.; aversion to fat under any quantity of remedies, but not under Lachesis, and still it was the remedy which gave the only relief.

